# MUSTN1 and FABP3 interact to regulate adipogenesis and lipid deposition

**DOI:** 10.1016/j.jlr.2025.100804

**Published:** 2025-04-15

**Authors:** Yu Fu, Xin Hao, Jingru Nie, Hongliang Zhang, Peng Shang, Bo Zhang, Hao Zhang

**Affiliations:** 1 State Key Laboratory of Animal Biotech Breeding, China Agricultural University, Beijing, China; 2 Sanya Research Institute of Chinese Academy of Tropical Agricultural Sciences, Hainan, China; 3 Coconut Research Institute, Chinese Academy of Tropical Agricultural Sciences, Hainan, China; 4 College of Animal Science, Xizang Agricultural and Animal Husbandry College, Linzhi, China

**Keywords:** MUSTN1, FABP3, adipogenesis, lipid deposition, PI3K/AKT

## Abstract

Lipid deposition is related to agricultural animal production and human health, and elucidating its molecular regulatory mechanisms is a topic of interest and a challenge in current scientific research. Musculoskeletal embryonic nuclear protein 1 (MUSTN1) regulates growth and development, including muscle tissue; however, its role in fat deposition remains unknown. Thus, our objective was to determine this role. Our new findings were as follows: *MUSTN1* was highly expressed in the fat tissue of pigs with strong adipose deposition capacity; functionally, *MUSTN1* promoted the proliferation and adipogenic differentiation of porcine and mouse preadipocytes. *MUSTN1* knockout mice were protected against HFD-induced obesity, hepatic steatosis, and insulin resistance; and fatty acid binding protein 3 was identified as an interacting protein of MUSTN1, which facilitated preadipocyte proliferation and differentiation by activating the phosphatidylinositol 3 kinase/AKT signaling pathways. This study reveals a positive regulator for fat development, which suggests a novel approach for studying obesity and animal genetic improvement through the modulation of *MUSTN1* expression.

Enthusiasm for research on the physiology and developmental biology of adipose tissues is increasing because adipocytes are essential regulators of whole-body energy homeostasis, lipid deposition affects important economic traits such as reproduction and meat production in agricultural animals, and obesity has become a public health hazard worldwide and its incidence is increasing (1, 2). Adipocytes are divided into two types based on their structure and function: white adipose tissue (WAT), which stores excess energy, and brown adipose tissue, which functions as an energy source. The main feature of obesity is excessive WAT accumulation, which manifests as an increase in the size or number of fat cells (3). Excess fat tissue can lead to cardiovascular disease and type 2 diabetes (4). Adipogenesis is a complex physiological process regulated by many genes, non-coding RNAs, growth factors, and signaling pathways (5, 6). Adipose tissue development originates in the embryonic mesoderm and depends on the maturation of adipose cells in two stages: proliferation and differentiation (7). This process involves the transformation of mesenchymal stem cells into preadipocytes and the induction of preadipocytes into mature adipocytes, which provide space for the accumulation of lipid droplets (8, 9). Fat deposition is influenced by genetics, species, breed or diet, sex, and environmental factors, with genetic factors dominating (10, 11). Exploring this process and its regulatory mechanisms is of substantial significance in agriculture and human medicine.

Musculoskeletal embryonic nuclear protein 1 (MUSTN1) was originally identified in a rat femur fracture model and is believed to be specifically expressed only in skeletal muscles and tendons (12). *MUSTN1* is highly expressed in muscle tissues of fast-growing species, and this phenomenon has been found in several species, such as chickens (13, 14), yak (15), mink (16), donkeys (17), sheep (18) and ducks (19), using RNA-seq or mRNA expression analysis. Subsequent studies have shown that *MUSTN1* is expressed in bones (20), cartilage (21), and satellite cells (22). Thus, current studies on the function of the *MUSTN1* gene mainly focus on skeletal and muscle development. *MUSTN1* is required for craniofacial chondrogenesis (20), chondrocyte proliferation, and differentiation in vitro (21). Reducing the expression of *MUSTN1* inhibits myoblast differentiation and fusion of myoblasts significantly (23, 24). *MUSTN1* ablation in skeletal muscle results in increased glucose tolerance, affecting whole-body glucose homeostasis (25). We found that *MUSTN1* was highly expressed in the adipose tissue of pigs and mice, and *MUSTN1* deletion resulted in reduced subcutaneous fat content in the legs of mice (26). Thus, we speculate that *MUSTN1* has a regulatory effect on adipogenesis and fat deposition. However, the function of this gene in fat development and its specific mechanism of action remains poorly understood.

Pigs are economically important animals that exhibit a high degree of similarity to humans in anatomy, physiology, biochemistry, pathology, and pharmacology; thus, pigs are ideal biological models for studying fat development and obesity-related diseases. In this study, we investigated the effects and regulatory pathways of porcine *MUSTN1* on preadipocyte proliferation and differentiation. A *MUSTN1* knockout (KO) mouse model on a high-fat diet (HFD) was used to explore the gene's effects on fat deposition, glucose metabolism, and insulin sensitivity. The results revealed a new biological function in fat deposition for *MUSTN1* and provided new ideas and targets for improving the economic benefits of animal husbandry and preventing obesity-related diseases.

## Materials and Methods

### Cell culture, adipogenic differentiation, plasmid construction, and siRNA synthesis

3T3-L1 and primary preadipocytes (Type Culture Collection of the Chinese Academy of Sciences) were cultured in Dulbecco's modified Eagle medium/nutrient mixture F-12 (DMEM/F12, Gibco) with 10% fetal bovine serum (Gibco) and 1% penicillin/streptomycin (Gibco) at 37°C in a 5% CO_2_ incubator. For cell differentiation experiments, approximately 3 × 10^5^ cells were evenly seeded into 24-well plates through cell counting, and transfection was performed once the cells reached 90% confluence. The cells reached near full confluence after six hours post-transfection, and then were differentiated in DMEM supplemented with 5 μg/ml insulin, 1 μM dexamethasone, and 0.5 mM 3-isobutyl-1-methylxanthine for 2 days (all from Sigma-Aldrich). The medium was maintained with 5 μg/ml insulin and replaced every 2 days. The specific primer sequences for the construction of the pCDH-*MUSTN1*-pig, pCDH-*MUSTN1*-mouse, pcDNA3.1-*MUSTN1*-mouse-3×FLAG, and pcDNA3.1-*FABP3*-mouse-3×FLAG overexpression vectors are shown in supplemental Table S1. The sequences of the mouse *MUSTN1* siRNAs are listed in supplemental Table S2. The cells were transfected per the instructions of Lipofectamine 2000 (Invitrogen).

### Isolation of primary preadipocytes

Primary preadipocytes were isolated from the subcutaneous fat of 6-week-old male mice and 7-day-old boars. Dissecting adipose tissues was followed by enzymatic digestion to dissociate the cells using 2 mg/ml collagenase type I (Sigma-Aldrich) and 2.5 U/ml dispase (Roche Applied Science) at 37°C for 40 min. The digested tissue was filtered using a 70-μm filter membrane (Biosharp) and then centrifugation at 1,000 *g* for 5 min at 4°C to separate the preadipocytes, which were subsequently cultured in DMEM/F12 for further studies.

### RNA extraction and mRNA level measurement

Total RNA was extracted from cells and tissues using the TRIzol reagent (Invitrogen). Briefly, trizol was added to the sample for ice bath or homogenization until no tissue or cell debris was visible, then the RNA was separated from the protein and DNA using phenol-chloroform extraction, the RNA was precipitated using isopropyl alcohol, and the RNA precipitate was collected by centrifugation (4°C, 12,000 rpm, 15 min). Extracted RNA was then reverse transcribed using the FastQuant Reverse Transcriptase Kit (TIANGEN). The mRNA expression levels of the target genes were analyzed using semi-quantitative real-time polymerase chain reaction (PCR) and quantitative RT-PCR using specific primers (supplemental Table S3). β-actin was used as the housekeeping gene to normalize the expression level using the 2^-ΔΔCt^ method (27).

### Pigs and *MUSTN1*-KO mice

Tibetan pigs (TP) with strong fat deposition ability and Yorkshire pigs (YY) with low fat deposition ability were grown using standard food and water at the farm of Xizang Agricultural and Animal Husbandry University. Samples from the embryos of two pregnant sows were collected 60 days post-insemination. The *MUSTN1*-KO mouse line (C57BL/6N background) with a 2,207 bp deletion was generated by Cyagen using gRNAs (supplemental Table S4). Genotypes were determined using PCR from tail DNA with primers listed in supplemental Table S4. Wild-type (WT) and *MUSTN1*-KO mice had amplicons of 606 and 409 bp, respectively. Mice mate to produce offspring in a 3:1 ratio of males to females, and male mice were randomly selected. They were housed in a pathogen-free facility with controlled temperature (22 ± 1°C) and humidity (60 ± 10%) and a 12-h light/dark cycle, where they were fed water ad libitum and a normal feeding diet (NFD) or HFD (60 kcal%; Research Diets). The HFD group was fed an HFD from 2 to 5 months of age. The whole-body fat content of HDF-fed mice at 5 months old was measured using magnetic resonance imaging (MRI) at the Institute of Laboratory Animals Science, CAMS & PUMC.

### Experimental procedures

All procedures involving animals were conducted according to the National Research Council Guide for the Care and Use of Laboratory Animals, with the experimental protocols receiving approval from the Institutional Animal Care and Use Committee at China Agricultural University (permit number: AW80203202-1-1).

### Cell proliferation measurement

5-ethynyl-2-deoxyuridine (EdU) and cell counting kit-8 (CCK8) were used to measure cell proliferation. After mixing and counting, approximately 1 × 10^4^ cells were seeded in 96-well plates. Transfection was performed when cell confluence reached 40% to 60%, and cell proliferation was assessed 24 h after transfection. For the EdU (Ribobio) assay, cells incubated with 50 μM EdU for 2 h were fixed with 4% paraformaldehyde and permeabilized with Triton X-100. Subsequently, the cells were incubated with Apollo staining reaction solution and Hoechst stain at 25°C in the dark for 30 min each. Finally, the stained cells were observed and imaged under a fluorescence microscope (Leica image analysis system, Model Q500MC). For the CCK8 (Beyotime) assay, the cells were replaced with a medium containing 10% CCK8 and incubated at 37°C for 1 h in the dark. Absorbance was measured at 450 nm using a microplate reader (BioTek).

### Adipose differentiation staining

Oil Red O (Sigma-Aldrich) and BODIPY (Sigma-Aldrich) were used to stain lipid droplets, reflecting the adipose differentiation ability. Fixed cells were incubated with Oil Red O for 30 min and 60% isopropyl alcohol for 10 s or stained with 0.1% BODIPY in PBS for 30 min. All stained cells were imaged using a microscope (ZEISS) or a fluorescence microscope (Leica). The optical density in the Oil Red O assay was measured at 510 nm using a spectrophotometer (Biotek).

### Hematoxylin-eosin (HE) staining

HE staining was performed on fat and liver tissues collected from HFD-fed mice to observe the histological morphology. After fixation in 4% paraformaldehyde, the tissue samples were cut into sections via routine paraffin histology and stained with HE. The sections were examined under an inverted microscope (ZEISS).

### Glucose and insulin tolerance tests (GTT, ITT)

GTT and ITT were conducted by injecting 20% glucose solution (2 g/kg body weight) or insulin (0.75 U/kg body weight) intraperitoneally after a 16 h or 4 h fast, respectively. Five-month-old HFD-fed male mice were used in these two assays, and their tail vein blood samples were used to measure glucose within 2.5 h post-injection.

### Western blot

Preadipocytes and fat tissues were lysed in RIPA buffer (Beyotime). The protein was resolved on SDS-PAGE and transferred to a PVDF membrane. Next, the membrane was blocked in QuickBlock buffer (Beyotime) for 30 min at 25°C and incubated with primary antibodies at 4°C overnight. Proteins were incubated with primary antibodies against anti-MUSTN1 (ABD115, 1:500, Sigma-Aldrich), anti-CEBPα (D56F10, 1:1000, CST), anti-PPARγ (sc7273, 1:200, Santa Cruz Biotechnology), anti-FABP3 (A5312, 1:1000, ABclonal), anti-AKT (9272, 1:2000, CST), anti-p-AKT-Ser473 (4060, 1:1000, CST), anti-PI3K (4249, 1:1000, CST), anti-p-PI3K (13857, 1:1000, CST) and anti-βactin (4970, 1:1000, CST), and anti-FLAG (20543-1-AP, 1:1000, Proteintech). The membranes were incubated with horseradish peroxidase-conjugated secondary antibodies (1:5000, Solarbio) for 1 h at 25°C. Protein expression was quantified using Gelpro32 software (Media Cybernetics).

### Co-immunoprecipitation (Co-IP)

Cells were transfected with pcDNA3.1-*MUSTN1*/*FABP3*-3×FLAG and an empty vector for 48 h and then lysed on ice with lysis buffer (RM00022, ABclonal) supplemented with a protease inhibitor cocktail (RM02916, ABclonal). Cells collected via centrifugation at 2000 rpm for 5 m were combined with 20 μl anti-FLAG magnetic beads. These beads were allowed to rotate continuously with the lysate at 4°C overnight. After three to five washes with lysis buffer, the solution was magnetically separated. The mixture supplemented with protein loading buffer was boiled for 10 min, and Western blot analysis was conducted to ascertain the enrichment of prey proteins in the final immunocomplex supernatant.

### Immunoprecipitation mass spectrometry (IP-MS)

For IP-MS assays, the antigen-antibody-magnetic bead complex was obtained as described above. The immunoprecipitation was verified using silver staining and Western blot, then samples treated with decolorizing enzyme were subjected to RIGOL L-3000 high-performance liquid chromatography system for mass spectrometry data collection. The mass spectral data were searched with Proteome Discoverer 2.4 software.

### Immunofluorescence staining

The cells were fixed with 4% paraformaldehyde and then treated with 0.1% Triton X-100. Then the blocked samples were incubated with the primary antibody at 4°C overnight, and the nuclei were stained by DAPI (Roche) after the second antibody (SA00013-2 and SA00013-3, 1:100, ABclonal) incubated for 1 h. Finally, the fluorescence signals were observed under a fluorescence microscope (Leica). The primary antibodies used were as follows: anti-FLAG (20543-1-AP, 1:100, Proteintech) and anti-FABP3 (A5321, 1:50, ABclonal).

### Statistical analysis

Data are presented as means ± standard deviation. For the purpose of analyzing statistical significance, Student's t tests were employed for pairwise comparisons between two groups, and a one-way ANOVA with Tukey's post hoc test was utilized for comparisons across multiple groups. Statistical significance was evaluated using GraphPad Prism v. 6.0 (GraphPad Software), and *P* < 0.05 was considered statistically significant. Data are representative of three or more independent experiments.

## Results

### MUSTN1 facilitated adipogenesis in pigs

**Fig. 1:**
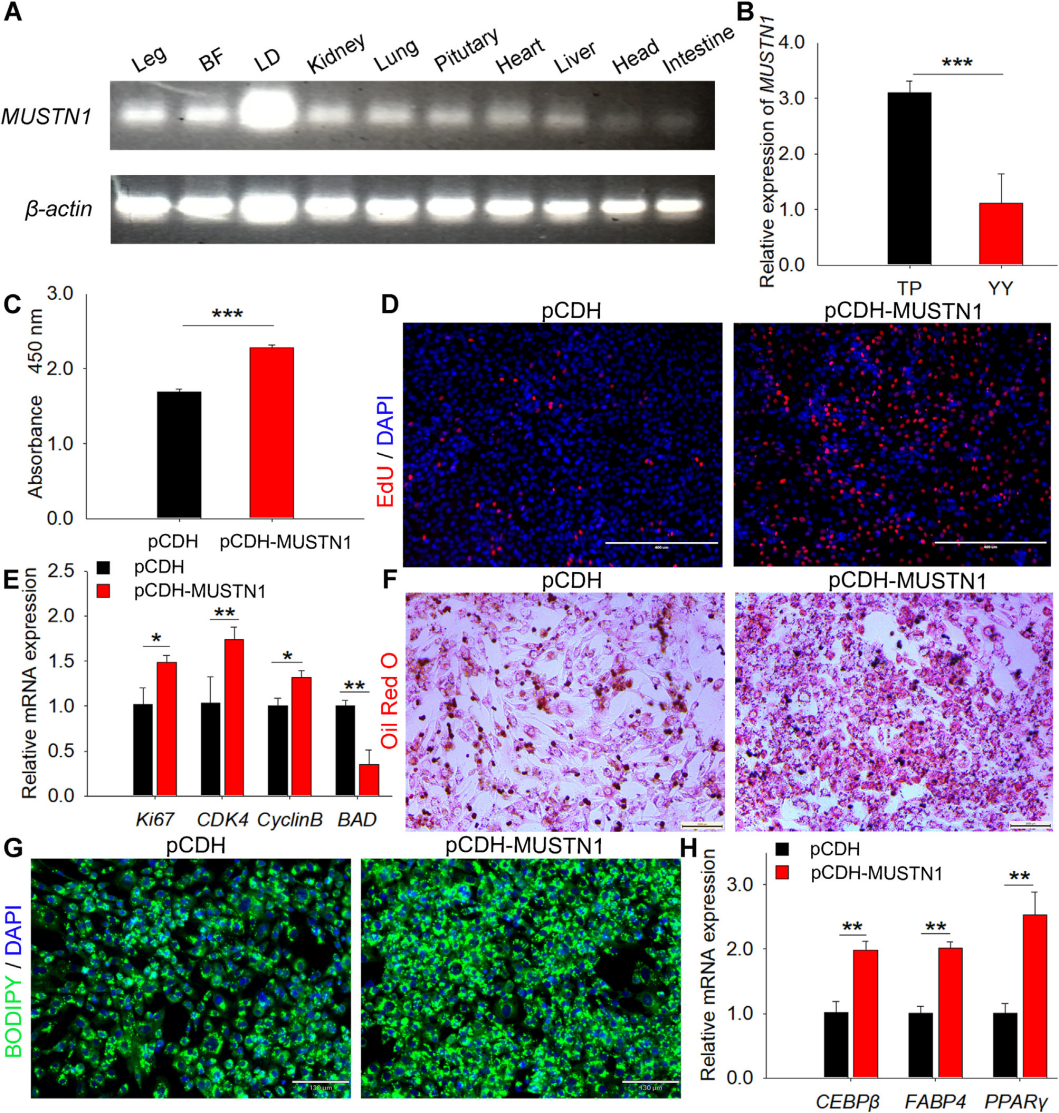
*MUSTN1* participates in the fat deposition of pigs. A: SqRT-PCR analysis of *MUSTN1* expression in various tissues of 60-day-old TP embryos, n = 6. BF, back fat; LD, *longissimus dorsi*. B: mRNA expression level of *MUSTN1* in the back fat tissues of embryonic TP and YY pigs. TP, Tibetan pig (n = 6); YY, Yorkshire (n = 6). CCK8 analysis (C) and EdU staining (D) of porcine adipocytes transfected with pCDH and pCDH- *MUSTN1*. EdU is shown in red and nuclei in blue (DAPI), scale bar = 400 μm. E: mRNA expression levels of proliferation (*Ki67*, *CDK4*, and *Cyclin B*) and apoptosis marker (*BAD*) genes. F: Representative photographs of Oil Red O staining in primary pig muscle cells differentiated for 8 days, scale bar = 200 μm. G: BODIPY staining for lipid droplet, scale bar = 130 μm. H: mRNA expression levels of adipogenic differentiation markers (*CEBPβ*, *FABP4*, and *PPARγ*). The data represent the mean ± SD of at least three independent experiments. The asterisk indicates a significant difference based on the Student's *t* test, ∗∗: *P* < 0.01, ∗∗∗: *P* < 0.001.

The expression pattern of the *MUSTN1* gene was investigated in 60-day-old embryonic TP pigs using qPCR and we found that it was widely expressed in various tissues, with high expression in *longissimus dorsi*, leg muscle, and back fat tissues (Fig. 1A). The expression level of *MUSTN1* in TP pigs that had high fat deposition ability was significantly higher than that in YY pigs that had low fat deposition ability (Fig. 1B), indicating a positive correlation between its expression and fat deposition capacity in pigs. To examine whether *MUSTN1* regulates fat deposition, we constructed a *MUSTN1* overexpression vector and transfected it into isolated primary porcine adipocytes (supplemental Fig. S1A). The results showed that overexpressing *MUSTN1* increased the absorbance value of cells after CCK8 treatment (Fig. 1C), the number of proliferating cells labeled with EdU (Fig. 1D), and the expression of proliferation marker genes (*Ki67, CDK4* and *Cyclin B*) (Fig. 1E) and downregulated the mRNA level of apoptosis gene *BAD* (Fig. 1E). Additionally, fat droplets labeled with Oil Red O (Fig. 1F) and BODIPY (Fig. 1G) staining were evaluated after *MUSTN1* overexpression, accompanied by upregulation of adipogenic differentiation marker genes (CCAAT/enhancer binding protein beita, *CEBPβ*; fatty acid binding protein 4, *FABP4*; and peroxisome proliferator-activated receptor gamma, *PPARγ*) (Fig. 1H). These results suggest that *MUSTN1* promotes adipocyte proliferation and differentiation, regulating fat development in pigs.

### MUSTN1 positively regulated proliferation and differentiation of 3T3-L1

**Fig. 2:**
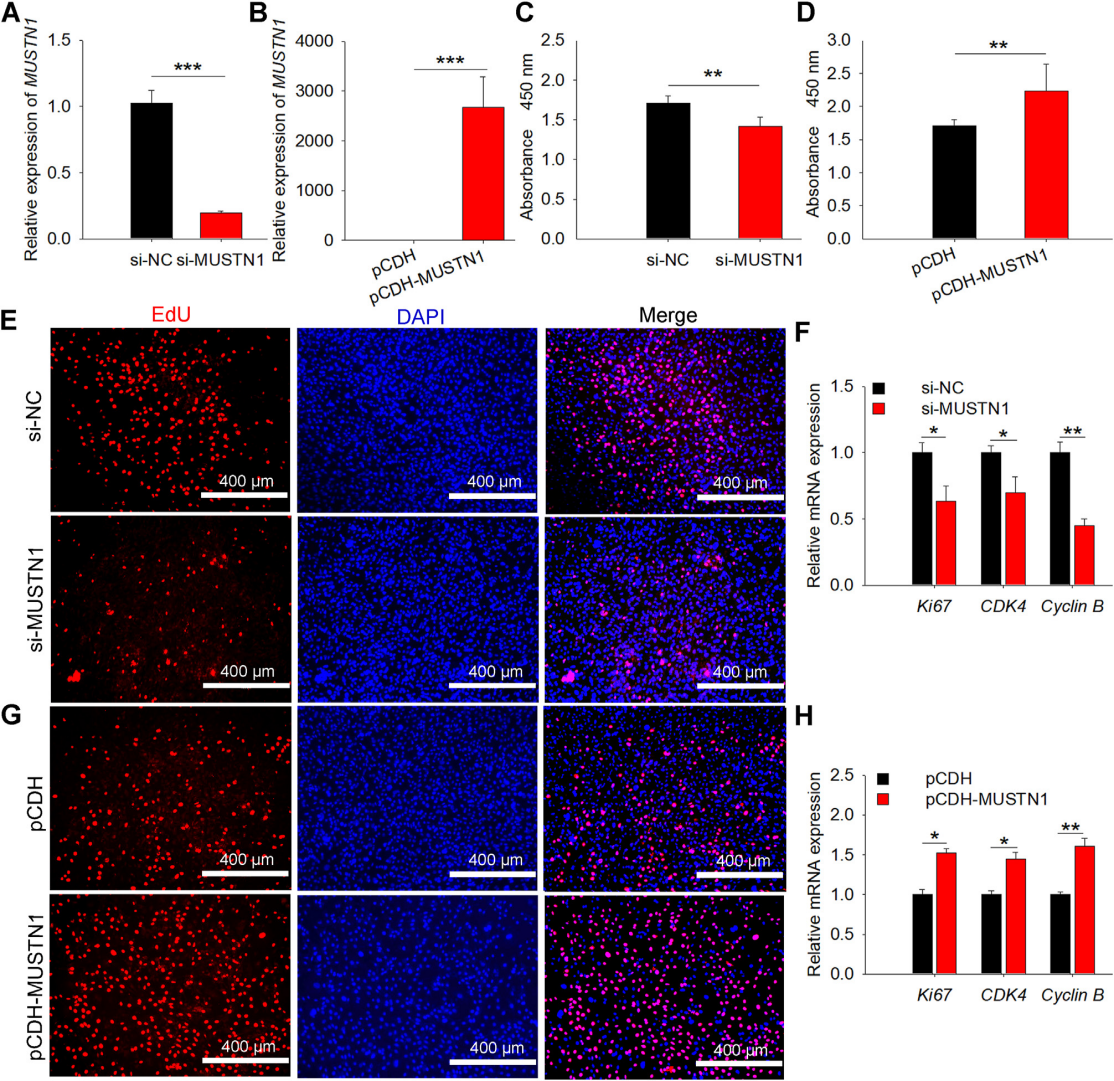
*MUSTN1* promotes the proliferation of 3T3-L1 cells. Detection of interference (A) and overexpression (B) efficiency of *MUSTN1* gene. CCK8 assay of 3T3-L1 cells transfected with si-*MUSTN1* (C) or pCDH-porcine-*MUSTN1* (D). EdU staining (E) and mRNA expression levels of proliferation-related genes (F) following RNAi transfection. Nuclei were stained with DAPI, NC: negative control, n = 3 in each group, scale bar = 400 μm. EdU staining (G) and mRNA expression analysis (H) for *MUSTN1* overexpressed cells. scale bar = 400 μm. The data represent the means ± SD of three independent experiments. The asterisk indicates a significant difference based on the Student's *t* test, ∗: *P* < 0.05, ∗∗: *P* < 0.01, ∗∗∗: *P* < 0.001.

**Fig. 3:**
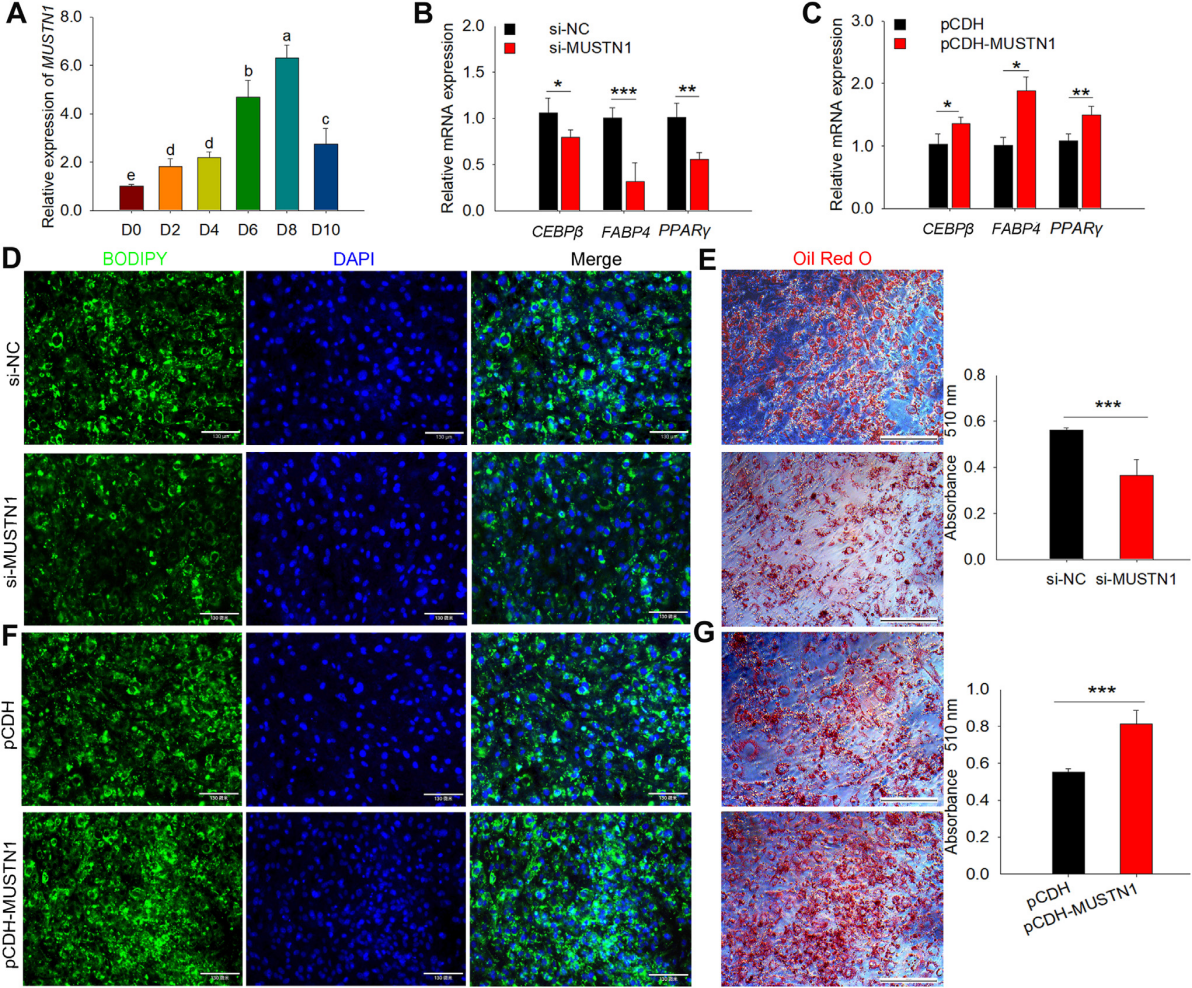
*MUSTN1* accelerates the adipogenic differentiation of 3T3-L1 cells. A: qRT-PCR results showed the expression profiles of the *MUSTN1* gene during differentiation. D0-D10 indicates lipogenic differentiation for 0–10 days. Different letters represent significant differences between groups. B and C: The mRNA expression of lipogenic differentiation marker genes quantified by qRT-PCR. D, E BODIPY and Oil red O staining for lipid droplet in *MUSTN1* knockdown 3T3-L1 cells. The cells were incubated in differentiation medium for 8 days. Oil Red O labeled-fat droplets were eluted with isopropyl alcohol, and absorbance was measured at 510 nm (right bar chart). BODIPY, scale bar = 130 μm. Oil red O, scale bar = 300 μm. NC: negative control. F and G: Representative photographs of BODIPY and Oil red O staining in *MUSTN1* overexpressed cells. BODIPY, scale bar = 130 μm. Oil red O, scale bar = 300 μm. The data represent the means ± SD of three independent experiments. The asterisk indicates a significant difference based on the Student's *t* test, ∗: *P* < 0.05, ∗∗: *P* < 0.01, ∗∗∗: *P* < 0.001.

The 3T3-L1 cell line has not only stable passage but also good specificity for adipocyte differentiation, making it an internationally recognized cell model for the study of adipocyte metabolism. Therefore, we further explored the biological function of *MUSTN1* in 3T3-L1 cells by using knockdown (Fig. 2A and supplemental Fig. S1B) or overexpression (Fig. 2B and supplemental Fig. S1C). CCK8 analysis revealed that the absorbance value after *MUSTN1* knockdown was reduced compared with that of the control group (Fig. 2C), and the opposite effects were observed after *MUSTN1* overexpression (Fig. 2D). *MUSTN1* knockdown also decreased the number of EdU-positive cells (Fig. 2E) and the expression of proliferation marker genes (Fig. 2F). When *MUSTN1* was overexpressed, EdU incorporation (Fig. 2G) and mRNA levels of pro-proliferation genes (Fig. 2H) increased. Moreover, the expression levels of *MUSTN1* gradually increased during the period of 0–8 days of adipogenic differentiation and then decreased on day 10 of differentiation (Fig. 3A). Thus, we selected 8 days of adipose differentiation as the time point for subsequent differentiation experiments. *MUSTN1* knockdown downregulated the expression of adipogenic differentiation markers (Fig. 3B), and *MUSTN1* overexpression upregulated their expression (Fig. 3C). Compared with the control group, *MUSTN1* knockdown led to a decreased BODIPY- (Fig. 3D) or Oil Red O (Fig. 3E)-labeled lipid droplets. Elevated results in these two assays were observed in *MUSTN1-*overexpressed cells (Fig. 3F, G). These data indicate that *MUSTN1* plays a positive role in preadipocyte proliferation and differentiation in vitro, and these functions exhibit certain similarities and conservation with what we have found in pigs.

### MUSTN1-KO mice were protected against HFD-induced obesity, hepatic steatosis, and insulin resistance

**Fig. 4:**
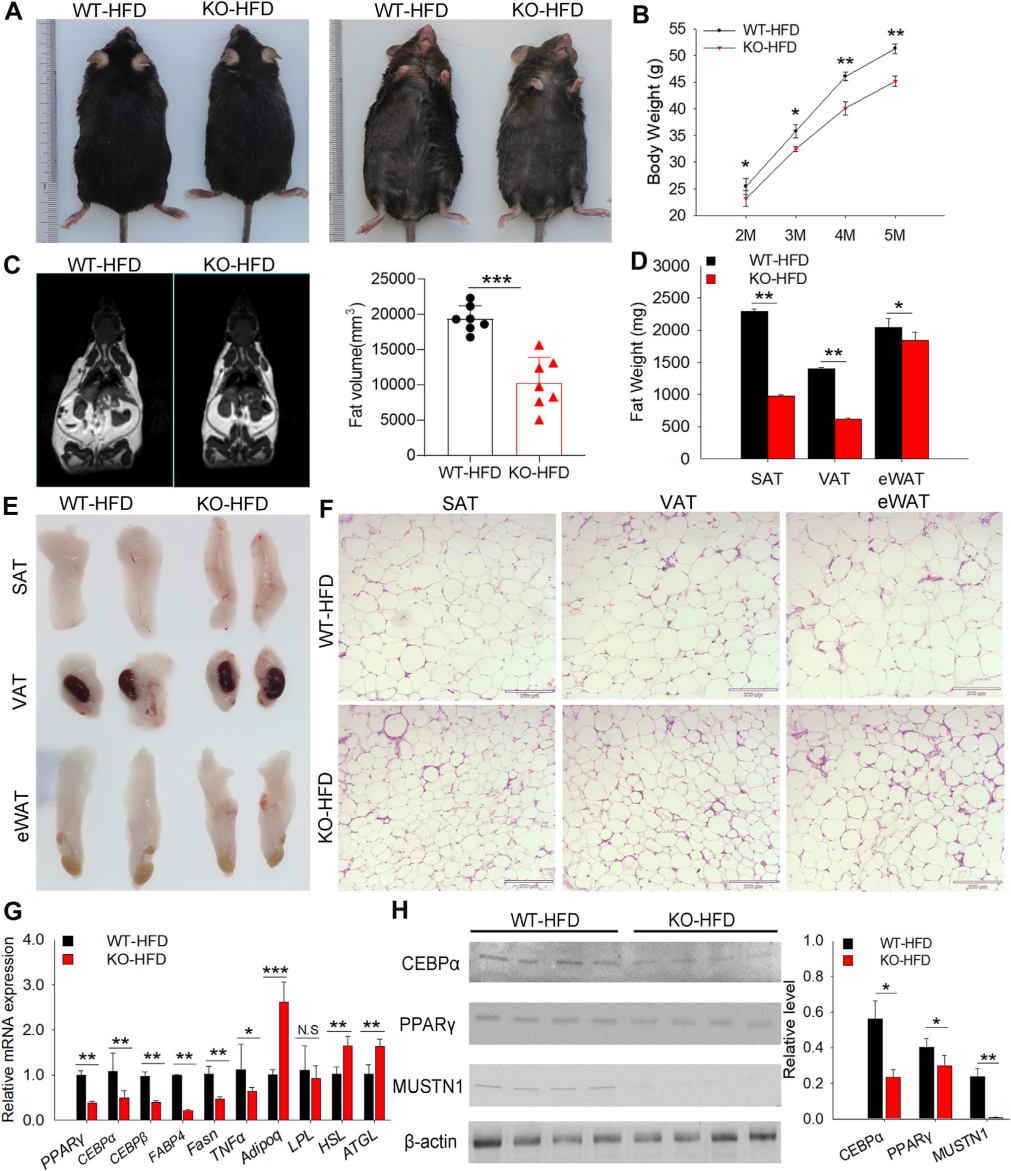
*MUSTN1* deficiency renders mice resistant to HFD-induced obesity. Representative gross morphology (A) and body weight curves (B) of whole bodies of wild-type (WT) and *MUSTN1* knockout (KO) mice at 20 weeks of age fed a high-fat diet (HFD) for 12 weeks; n > 10. C: Representative magnetic resonance images of the whole fat of HFD-fed WT and KO mice. Fat volumes are presented as mm3 (n = 7). D: Quantification of fat weight in HFD-fed mice (n = 6). SAT, subcutaneous adipose tissue; VAT, visceral adipose tissue; eWAT, epididymal white adipose tissue. E: Representative images of the fat pads of HFD-fed mice. F: Representative images of HE-stained sections of SAT, VAT, and eWAT from HFD-fed mice; scale bar = 210 mm. G: mRNA expression level of lipogenic-related genes (*PPARγ*, *CEBPα*, *CEBPβ*, *FABP4*, *Fasn*, and *TNFα*) and lipolysis-related genes (*Adipoq*, *LPL*, *HSL*, and *ATGL*) in fat tissues of HFD fed mice, n = 6. H: Western blot analysis of CEBPα and PPARγ levels in fat tissues of HFD-fed mice, n = 3. The data represent the mean ± SD. The asterisk indicates a significant difference based on the Student's *t* test, ∗: *P* < 0.05, ∗∗: *P* < 0.01, ∗∗∗: *P* < 0.001. N.S.: not significant.

We generated *MUSTN1*-KO mice to explore the in vivo function of *MUSTN1* in fat deposition. Under normal feeding diet conditions, the weights of subcutaneous, visceral, and epididymal white adipose tissues (SAT, VAT, and eWAT, respectively) were lower in male KO mice aged 2–4 months of age than in WT mice, even though both groups were healthy and born at the expected Mendelian ratio (supplemental Fig. S2A–C). However, the weight of the interscapular brown adipose tissue did not differ significantly between male WT and KO mice (supplemental Fig. S2A–C). And the differences in weight between WT and KO female mice were not significant for both white adipose tissue and brown adipose tissue (supplemental Fig. S2D). These findings indicate that *MUSTN1* mainly regulates the development of white fat in male mice. To better understand *MUSTN1* function in vivo, we subjected the mice to a HFD for 3 months to induce obesity. Intriguingly, *MUSTN1*-KO mice exhibited a smaller body size (Fig. 4A) and lower body weight and length (Fig. 4B) than WT controls. However, no significant difference was observed in food intake (supplemental Fig. S2E). MRI revealed significantly reduced fat volume in *MUSTN1*-KO mice compared with WT mice (Fig. 4C). *MUSTN1*-KO mice had decreased fat weight (Fig. 4D), fat mass (Fig. 4E), and adipocyte area (Fig. 4F). Corresponding to these phenotypes, the mRNA expression level of the adipocyte differentiation-, lipogenic- and adipokine-related genes, *PPARγ*, *CEBPα*, *CEBPβ*, and *FABP4*, and fatty acid synthase (Fasn) and tumor necrosis factor alpha (*TNFα*), were markedly downregulated in the adipose tissue of *MUSTN1*-KO mice (Fig. 4G). The mRNA expression levels of the lipolysis-related genes, adiponectin (*Adipoq*), lipolysis (*LPL*), hormone-sensitive lipase (*HSL*), and adipose triglyceride lipase (*ATGL*), were upregulated (Fig. 4G). The protein levels of CEBPα and PPARγ were consistent and corresponded to the mRNA levels (Fig. 4H). These results revealed that *MUSTN1*-KO mice exhibited resistance to obesity after HFD feeding.

**Fig. 5:**
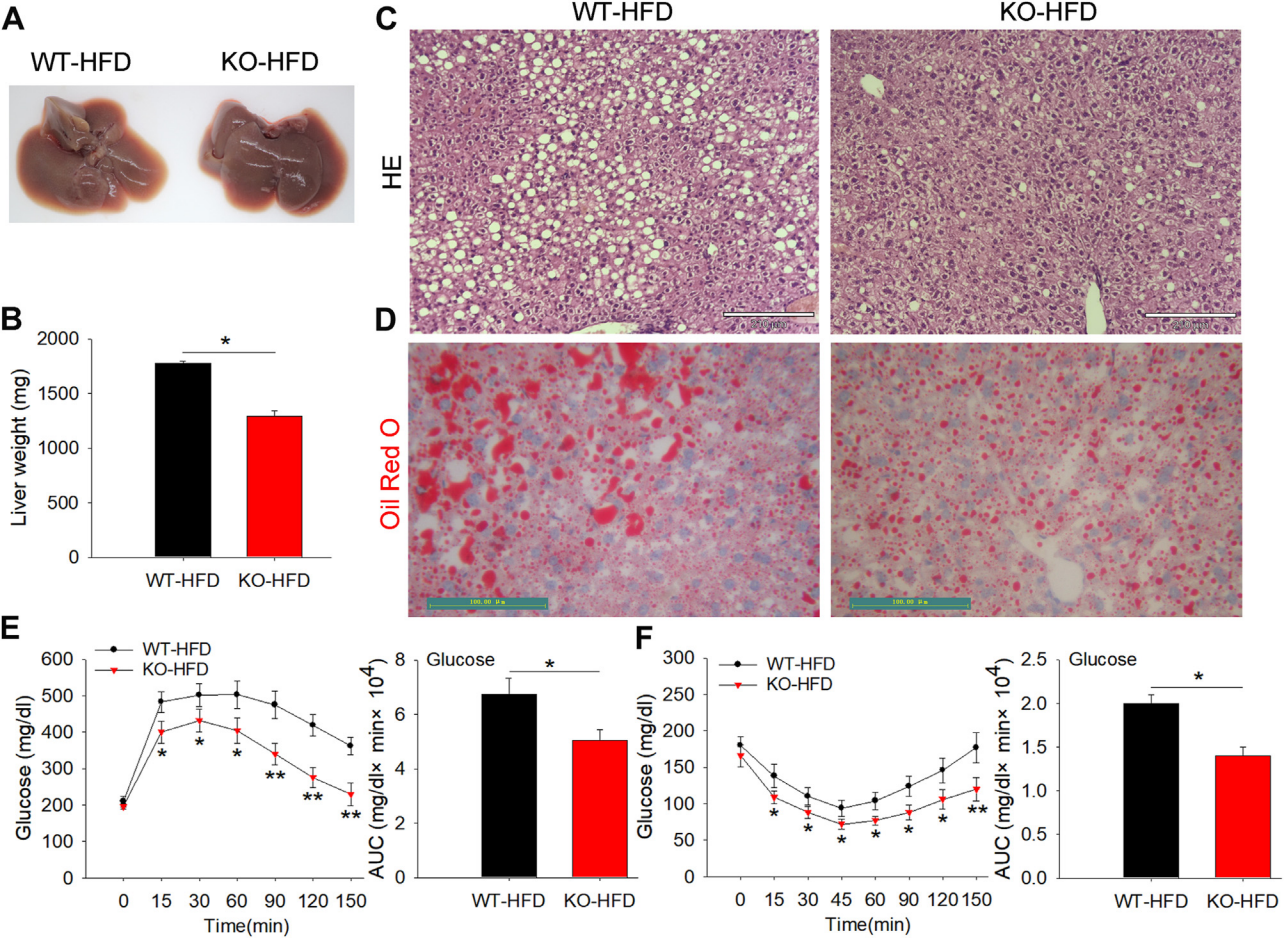
*MUSTN1*-KO mice were protected against HFD-induced hepatic steatosis and insulin resistance. A: Representative images of the liver in HFD-fed WT and *MUSTN1*-KO mice. B: Liver weight of HFD-fed WT and KO mice at 20 weeks of age fed an HFD for 12 weeks (n = 6). Representative images sections of the liver stained with HE (C) and Oil Red O (D). HE, scale bar = 210 μm; Oil Red O, scale bar = 100 μm. Glucose tolerance (E) and insulin tolerance (F) tests in HFD-fed mice (n > 4). Bar charts show the area under the curve (AUC) for glucose. The data represent the mean ± SD. The asterisk indicates a significant difference based on the Student's *t* test, ∗: *P* < 0.05, ∗∗: *P* < 0.01.

The liver, the primary site of fat synthesis, plays a crucial role in fat metabolism. During dissection, we observed that the livers of *MUSTN1*-KO mice were a darker color (Fig. 5A) and had a lower weight (Fig. 5B) and fewer fat droplets stained with HE (Fig. 5C) and Oil Red O (Fig. 5D) than the livers of the WT mice. This result indicates that *MUSTN1*-KO mice can reduce HFD-induced ectopic fat deposition in the liver. Obesity is often associated with abnormal insulin levels and disrupted glucose metabolism. To confirm whether these phenomena also exist in WT and *MUSTN1*-KO mice treated with a HFD, we examined serum glucose levels after the injection of glucose or insulin at different time points. The GTT results showed that the glucose concentration increased rapidly after the intraperitoneal injection of glucose in each group and gradually decreased after reaching a peak at 30 min. In *MUSTN1*-KO mice, glucose concentration and area under the curve were significantly lower than those in WT mice (Fig. 5E), suggesting that mice in the *MUSTN1* deficiency group had better glucose tolerance than WT mice. ITT results showed that the concentrations of glucose and AUC in *MUSTN1*-KO mice after insulin injection were lower than those in WT mice (Fig. 5F), indicating that insulin sensitivity determined by ITT was significantly improved in *MUSTN1-*KO mice. Similarly, blood biochemical results showed lower levels of glucose metabolism in HFD-fed *MUSTN1*-KO mice than those in WT mice (Table 1). Notably, the levels of most lipid profiles (total cholesterol and high/low density lipoprotein) and adipokines (TNF-α, IL-6, and resistin) also significantly declined in the *MUSTN1*-KO mice. However, triglyceride, non-esterified fatty acid, and adiponectin levels did not differ significantly from those in WT mice (Table 1). These results, in line with our IPGTT and ITT data, showed that *MUSTN1*-KO mice were protected against HFD-induced insulin resistance.

**Table 1:** Serum metabolite and adipokine levels in HFD-fed mice

Parameters	WT	KO	*P* Value
Glucose Metabolism			
Glucose (mmol/L)	6.60 ± 1.64	5.25 ± 0.56	0.02^a^
Insulin (uIU/ml)	13.02 ± 1.88	10.17 ± 0.53	0.01^a^
Lipid Profile			
Total cholesterol (mmol/L)	1.62 ± 0.13	1.13 ± 0.24	0.01^a^
Triglyceride (mmol/L)	0.30 ± 0.03	0.25 ± 0.03	0.07
Nonesterified fatty acid (mmol/L)	0.44 ± 0.10	0.42 ± 0.08	0.62
High density lipoprotein (mmol/L)	0.94 ± 0.09	0.63 ± 0.17	0.01^a^
Low density lipoprotein (mmol/L)	0.33 ± 0.03	0.19 ± 0.05	0.004^b^
Adipokine Levels			
TNF-α (pg/ml)	61.61 ± 9.82	36.60 ± 8.51	0.04^a^
IL-6 (pg/ml)	127.45 ± 8.49	80.51 ± 8.01	0.005^b^
Resistin (ng/ml)	23.66 ± 2.49	14.16 ± 1.70	0.0004^c^
Adiponectin (mg/ml)	12.86 ± 1.11	12.54 ± 1.11	0.002^b^

Data are represented as mean ± SD. n = 8 animals per group.

a *P* < 0.05.

b *P* < 0.01.

c *P* < 0.001.

### MUSTN1 deletion resulted in decreased proliferation and differentiation of preadipocytes

**Fig. 6:**
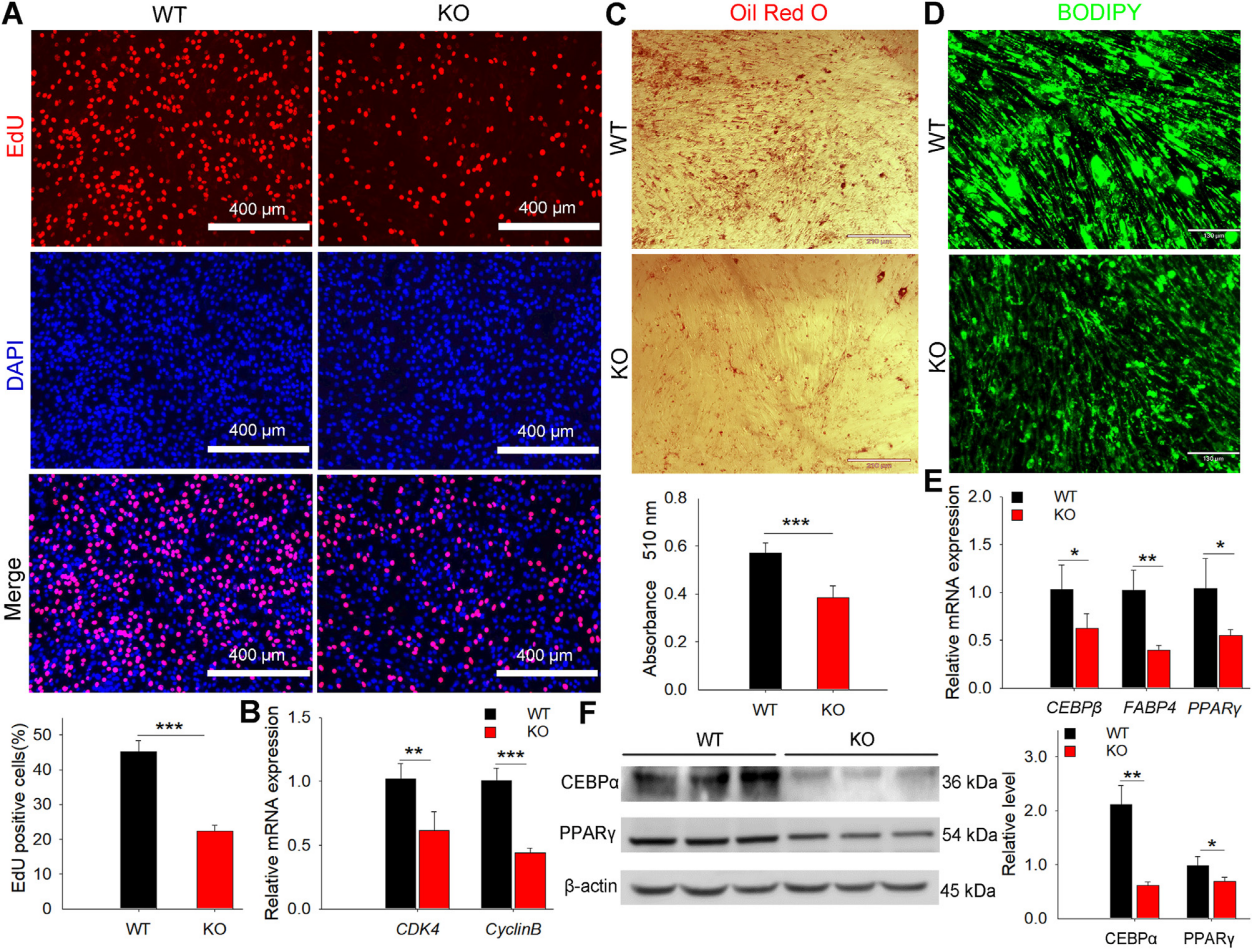
*MUSTN1* deletion restrains proliferation and differentiation of mouse primary preadipocytes. A: Cell proliferation was assessed by EdU staining. DAPI-stained nuclei are shown (scale bar = 400 μm). B: mRNA levels of *CDK4* and *Cyclin B* (proliferation markers) were quantified by qRT-PCR. C: Representative images of Oil Red O staining in control and KO cells that were allowed to differentiate for 4 days. Oil Red O-stained fat droplets are shown in red. Oil Red O-labeled fat droplets were eluted with isopropyl alcohol, and absorbance was measured at 510 nm (lower bar chart). D: BODIPY staining of differentiated WT and KO adipocytes. Green represents the BODIPY-labeled fat droplets. mRNA (E) and protein (F) expression levels of adipogenic differentiation marker genes in mouse primary preadipocytes. Data represent mean ± SD, n = 3. The asterisk indicates a significant difference based on the Student's *t* test, ∗: *P* < 0.05, ∗∗: *P* < 0.01, ∗∗∗: *P* < 0.001.

We isolated preadipocytes from the fat tissues of WT and *MUSTN1*-KO mice to determine the role of *MUSTN1*. *MUSTN1* deletion caused a reduction in the number of EdU-positive cells (Fig. 6A) and the expression of proliferation markers (Fig. 6B). Additionally, WT preadipocytes induced a greater accumulation of lipid droplets than those in the KO group without *MUSTN1* (Fig. 6C, D). Next, we evaluated whether the decreases in intracellular lipid contents in the *MUSTN1*-KO group were associated with decreased levels of PPARγ and CEBP, two major adipogenic proteins expressed in the early stage of adipogenesis (28). In support of the result we expected, quantitative analysis revealed that *MUSTN1*-KO induced the downregulation of mRNA (Fig. 6E) or protein (Fig. 6F) levels of adipogenic markers compared with those of the WT.

### MUSTN1 interaction with FABP3 regulated adipogenesis

**Fig. 7:**
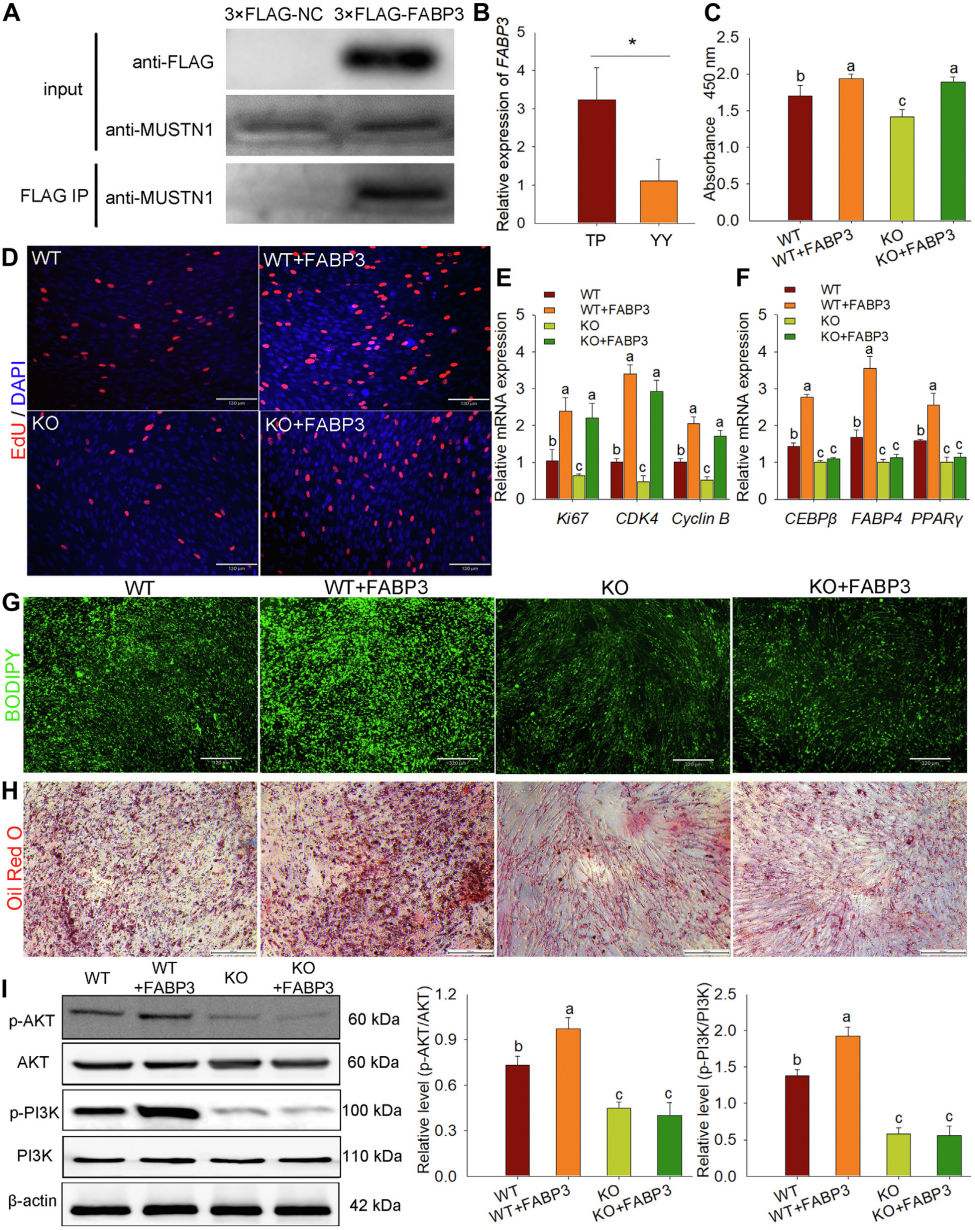
*MUSTN1* facilitates adipogenesis via the interaction with FABP3 and the phosphorylation of PI3K/AKT. A: Reciprocal co-immunoprecipitation analysis between FLAG-tagged mouse FABP3 in 3T3-L1 cells. IP, immunoprecipitation. B: mRNA expression level of *FABP3* in fat tissues of TP and YY pigs. CCK8 statistical analysis (C) and representative images of EdU staining (D), scale bar = 130 μm. Primary preadipocytes were isolated from WT and *MUSTN1*-KO mice and then transfected with or without FABP3 overexpression plasmid. E qRT-PCR analysis of proliferation (E) and differentiation (F) markers expression. Differentiation analysis using BODIPY (G) and Oil Red O (H). The cells were allowed to differentiate for 8 days. I: Western blot analysis of total and phosphorylated levels of PI3K/AKT was performed. Different letters represent significant differences between groups (*P* < 0.05). The data represent the mean ± SD. The asterisk indicates a significant difference based on the Student's *t* test, ∗: *P* < 0.05.

To explore the mechanism by which MUSTN1 regulates fat development, fatty acid binding protein 3 (FABP3) was identified as a potential interacting protein of MUSTN1 by using IP-MS. We immunoprecipitated MUSTN1 in 3T3-L1 cells transfected with 3×FLAG-*FABP3* and found that MUSTN1 and FABP3 were present in the precipitate and colocalized in the cytoplasm (Fig. 7A and supplemental Fig. S3A, B), confirming the interaction between MUSTN1 and FABP3. Similar to *MUSTN1*, the *FABP3* showed markedly higher expression in TP pigs with high fat deposition ability than that in YY pigs (Fig. 7B), suggesting a possible role of *FABP3* in fat deposition. We constructed a porcine *FABP3* overexpression plasmid and transfected it into mouse preadipocytes to investigate its function (supplemental Fig. S3C, D). The results showed that in the presence or absence of *MUSTN1*, *FABP3* overexpression increased the absorbance value (Fig. 7C), EdU-labeled cells (Fig. 7D), and the expression of pro-proliferation genes (Fig. 7E). However, due to the absence of *MUSTN1*, overexpression of *FABP3* did not rescue the inhibition of lipogenic differentiation (Fig. 7F–H). Notably, although both *MUSTN1* and *FABP3* promoted the proliferation and differentiation of adipocytes, the co-overexpression of both genes did not further enhance these promoting effects (supplemental Fig. S3E–J). Furthermore, considering the important role of the phosphatidylinositol 3-kinase/protein kinase B (PI3K/AKT) signaling pathway in cell proliferation and differentiation, we examined whether MUSTN1 and its interacting protein, FABP3, have regulatory effects on this signaling pathway. Western blot analysis revealed that FABP3 significantly increased the expression of phosphorylated PI3K and AKT without affecting the expression of total proteins, which was dependent on the presence of MUSTN1 (Fig. 7I). These findings imply that the promoted lipogenesis phenotype mediated by MUSTN1 is related to the activation of the PI3K/AKT signaling pathway.

## Discussion

Fat deposition is closely related to the growth and reproductive performance of agricultural animals and to human health. The study of fat development is of substantial value in the endeavor to alleviate various agricultural production problems and diseases caused by obesity. We found that *MUSTN1* was highly expressed in the adipose tissue of pigs with a strong adipose deposition capacity, revealing its potential regulatory role in adipose development. Therefore, this study mainly explored the biological functions of *MUSTN1* in lipogenesis and fat deposition through testing in vitro and in vivo, and explored the relevant regulatory pathways.

Lipogenesis is a complex process involving the early stage of adipocyte proliferation, characterized by the expansion of mitotic clones and the growth of preadipocytes to a state of cell differentiation after contact fusion. We confirmed that *MUSTN1* positively regulated the proliferation of porcine and mouse adipocytes by performing CCK8, EdU, and qPCR assays. Similarly, *MUSTN1* knockdown promoted apoptosis in chicken myoblasts (29). A critical feature of mature fat cells is the ability to accumulate lipids. When energy intake exceeds energy expenditure, excess energy is stored in fat tissue in the form of triglycerides (30). Oil Red O and BODIPY staining showed that *MUSTN1* promoted lipid droplet aggregation and upregulated the expression of adipocyte differentiation markers during lipogenesis. *PPARγ* is necessary for adipogenesis and to maintain a differentiated state (31). *PPARγ* upregulated the expression of adipocyte marker genes, including *adiponectin* and *FABP4* (8, 32). The upregulated expression of *FABP4* effectively promoted the terminal differentiation stage of adipocytes and accelerated their maturation, both of which play crucial roles in fatty acid storage, transport, and lipid signaling (33, 34). During lipogenesis, *CEBPβ* and *CEBPγ* are activated, upregulating the expression of *CEBPα* and *PPARγ*. *CEBPβ* and *CEBPγ* can also promote the expression of each other through a positive feedback loop and then affect the expression of downstream target genes to promote and maintain adipogenesis (35, 36). Our results showed that *MUSTN1* upregulated the mRNA and protein expression levels of *CEBPα* and *PPARγ*, further confirming that *MUSTN1* can promote the differentiation of preadipocytes into mature adipocytes. Obesity is one of five epidemic diseases worldwide that seriously endanger human health (1). Obesity in adults and children primarily manifested as hypertrophic and hyperplastic obesity, both of which are associated with the proliferation and differentiation of preadipocytes (37). Therefore, inhibiting adipocyte differentiation may be possible by altering cell differentiation conditions or regulating gene expression to prevent and treat obesity and related diseases.

We established a *MUSTN1*-KO mice model to investigate the relationship between *MUSTN1* and fat deposition. *MUSTN1*-KO mice were viable, healthy, and fertile, suggesting that *MUSTN1* is not crucial for normal development. We found that *MUSTN1* knockout mainly reduced WAT content (but not BAT) in male mice, and had no significant effect on female fat. Thus, this study focused on the relationship between *MUSTN1* and the development of WAT in male mice. We further established an obesity model in mice fed an HFD. Compared with the WT mice, the body weight and adipose tissue of the *MUSTN1*-KO mice were significantly reduced, but the organ weight (except for the liver) and food intake were not significantly affected, suggesting that the difference in body weight between the two mice is mainly caused by the difference in fat content, and the increase in fat mass in KO mice is not due to lower energy intake. Adipocyte size is determined by adipogenesis or the dynamic balance between the lipogenic and lipolytic processes (38). *HSL* and *ATGL* participate in lipolysis by acting on triglycerides and diglycerides to release free fatty acids (39). In the adipose tissue of the *MUSTN1*-KO group, our data indicated that the expression of lipogenesis markers was downregulated and that of lipolysis markers was upregulated. These results suggest that the reduced adipocyte size and fat content observed in *MUSTN1*-KO mice may be due to decreased adipogenic capacity and increased lipolytic capacity. The reduced proliferation and differentiation abilities of *MUSTN1*-KO adipocytes may further explain the fat-reducing phenotype at the cellular level. In addition, we recently found that *MUSTN1* knockout mice exhibited reduced muscle mass and fiber cross-sectional area, decreased exercise endurance, and delayed muscle regeneration (26). In view of the important role of *MUSTN1* in both muscle and fat development, we can further explore whether there is crosstalk between muscle development and fat deposition, and what role *MUSTN1* plays in it. The ideal model of tissue-specific knockout mice can also be used to further explore the specific effects of *MUSTN1* on muscle or fat development.

The liver is an important organ involved in fat metabolism. Excessive fat may cause abnormal liver metabolism and promote fat deposition. Fat hypertrophy leads to excessive feedback of fat storage signals, resulting in the disturbance of adipocytokines and a decreased ability of surrounding tissues to take up and utilize glucose (40). Excessive glucose continuously stimulates the secretion of large amounts of insulin by islet beta cells, which can induce the synthesis of triglycerides from fatty acids and glucose, resulting in the accumulation of triglycerides in the liver (41). Therefore, HFD mice exhibited ectopic adipose transfer and hepatic steatosis, and *MUSTN1*-KO mice alleviated the phenomenon of hepatic adipose deposition caused by an HFD (42).

Modern medicine regards obesity as a “metabolic disorder,” and the incidence of diseases caused by obesity, such as insulin resistance and fatty liver disease, is increasing annually (43, 44). Lipogenesis has important effects on various biological processes, such as insulin sensitivity, lipid metabolism, and inflammation (45). We observed that *MUSTN1*-KO enhanced glucose tolerance and insulin sensitivity in mice fed an HFD, protecting against HFD-induced insulin resistance. Kim *et al.* also reported that male mice with *Pax7*-Cre-driven muscle-specific *MUSTN1* deletion showed improved glucose tolerance at 2 months of age, but no difference was observed at any age in female mice (25). Our results and those of existing studies both indicated that MUSTN1 plays a role in whole-body glucose homeostasis. However, Ducommun *et al.* found that glucose tolerance showed no statistically significant differences between 2- and 3-month-old KO (Actb-Cre-driven muscle-specific Mustn1 deletion) and WT mice, although there was a trend toward improved glucose disposal in females (46). This difference may be due to slight differences in age, fasting period (5 h vs. 16 h), feed type (NFD vs. HFD), or the genetic model used (conditional vs. systemic KO).

Recent studies have shown that adipose tissue is not only an end-differentiated organ that provides energy storage but also an endocrine organ (47). Adipose tissue secretes various bioactive polypeptides known as adipokines, such as leptin, adiponectin, and resistin (48, 49, 50, 51). Adipokines regulate adipocyte differentiation, energy balance, metabolic homeostasis, and local inflammatory response through autocrine and paracrine pathways and play an important role in insulin resistance and chronic inflammation (52, 53). We found a reduced adipokine content in *MUSTN1*-KO mice compared with WT mice, which also explains the phenotype of reduced fat mass and improved insulin resistance from another perspective.

*FABP* participates widely in the transient storage and transport of fatty acids in cells (54). We found that MUSTN1 interacted with FABP3 to regulate adipogenesis. The literature has reported that *FABP3* is involved in the regulation of preadipocyte differentiation, fatty acid transport, and fat deposition in chicken (55) and dairy cattle (56). The expression of *FABP3* was increased during lipogenic differentiation (57). Overexpression of *FABP3* promoted adipocyte differentiation by upregulating adipogenic genes, such as *PPARγ* (57), and interference with *FABP3* led to a reduction in lipid droplets (58). Several studies have revealed a significant association between *FABP3* polymorphisms and intramuscular fat content in pigs (59, 60, 61, 62) and sheep (63, 64). Furthermore, *FABP3* methylation was associated with lipid, insulin, and blood pressure indicators, affecting metabolic syndromes in humans (65). However, the pathway or mechanism through which *FABP3* regulates fat deposition remains unclear.

Existing studies have demonstrated the involvement of PI3K in obesity-related biological processes (66). PI3K is a member of a unique and conserved family of enzymes responsible for protein phosphorylation. The insulin-mediated PI3K/AKT signaling pathway plays an important role in the fat cells of patients who are obese by regulating insulin resistance and glucose metabolism (67). The PI3K signaling pathway plays a key role in regulating preadipocyte differentiation, the inhibition of PI3K downregulates *PPARγ* expression, and blocks adipose differentiation (68). To further investigate whether the PI3K/AKT signaling pathway is involved in the regulation of adipogenesis *MUSTN1*, *MUSTN1*-deficient adipocytes were sampled to study relevant protein expression. Our data showed that MUSTN1 enhanced the phosphorylation of PI3K and AKT, promoting adipogenesis. Our research provides a basis for understanding the correlation between MUSTN1 and the PI3K/AKT signaling pathway in the regulation of lipidosis.

In summary, we report, for the first time, that *MUSTN1* regulates adipogenesis and fat deposition in *vitro* and in *vivo*. We confirmed that *MUSTN1* promoted the proliferation and lipogenic differentiation of porcine and mouse adipocytes. *MUSTN1*-KO mice fed an HFD exhibited reduced fat mass, improved hepatic adipose deposition, and insulin sensitivity. Mechanistically, MUSTN1 interacted with FABP3 and facilitated preadipocyte proliferation and differentiation by activating PI3K/AKT signals by upregulating the phosphorylation of PI3K/AKT. Our work revealed the critical role of *MUSTN1* in regulating adipogenesis by interacting with FABP3 and activating the PI3K/AKT signaling pathway, providing a theoretical foundation and a potential molecular target for improving animal performance and treating obesity-related diseases.

## Data availability

The data that support the findings of this study are available from the corresponding author upon reasonable request.

## Supplemental data

This article contains supplemental data.

## Conflict of interest

The authors declare that they have no conflicts of interest with the contents of this article.

## References

[1] Wolin K.Y., Carson K., Colditz G.A. (2010). Obesity and cancer. Oncologist.

[2] Yanovski S.Z., Yanovski J.A. (2011). Obesity prevalence in the United States--up, down, or sideways?. N. Engl. J. Med..

[3] Chen X., Zhao C., Xu Y., Huang K., Wang Y., Wang X. (2021). Adipose-specific BMP and activin membrane-bound inhibitor (BAMBI) deletion promotes adipogenesis by accelerating ROS production. J. Biol. Chem..

[4] Fukuda T., Hamaguchi M., Kojima T., Hashimoto Y., Ohbora A., Kato T. (2016). The impact of non-alcoholic fatty liver disease on incident type 2 diabetes mellitus in non-overweight individuals. Liver Int..

[5] Lopez-Pajares V. (2016). Long non-coding RNA regulation of gene expression during differentiation. Pflugers Arch..

[6] Tang Q.Q., Lane M.D. (2012). Adipogenesis: from stem cell to adipocyte. Annu. Rev. Biochem..

[7] Poulos S.P., Dodson M.V., Culver M.F., Hausman G.J. (2016). The increasingly complex regulation of adipocyte differentiation. Exp. Biol. Med. (Maywood).

[8] Farmer S.R. (2006). Transcriptional control of adipocyte formation. Cell Metab..

[9] Lefterova M.I., Lazar M.A. (2009). New developments in adipogenesis. Trends Endocrinol. Metab..

[10] Kempster A.J. (1981). Fat partition and distribution in the carcasses of cattle, sheep and pigs: a review. Meat Sci..

[11] Nematbakhsh S., Pei Pei C., Selamat J., Nordin N., Idris L.H., Abdull Razis A.F. (2021). Molecular regulation of lipogenesis, adipogenesis and fat deposition in chicken. Genes (Basel).

[12] Lombardo F., Komatsu D., Hadjiargyrou M. (2004). Molecular cloning and characterization of Mustang, a novel nuclear protein expressed during skeletal development and regeneration. FASEB J..

[13] Yin X., Fang W., Yuan M., Sun H., Wang J. (2023). Transcriptome analysis of leg muscles and the effects of ALOX5 on proliferation and differentiation of myoblasts in Haiyang yellow chickens. Genes (Basel).

[14] Gu S., Huang Q., Jie Y., Sun C., Wen C., Yang N. (2024). Transcriptomic and epigenomic landscapes of muscle growth during the postnatal period of broilers. J. Anim. Sci. Biotechnol..

[15] Ma X., Jia C., Chu M., Fu D., Lei Q., Ding X. (2019). Transcriptome and DNA methylation analyses of the molecular mechanisms underlying with longissimus dorsi muscles at different stages of development in the polled yak. Genes (Basel).

[16] Rong M., Xing X., Zhang R. (2024). Muscle transcriptome analysis of mink at different growth stages using RNA-seq. Biology (Basel).

[17] Yu J., Yang G., Li S., Li M., Ji C., Liu G. (2023). Identification of Dezhou donkey muscle development-related genes and long non-coding RNA based on differential expression analysis. Anim. Biotechnol..

[18] Vuocolo T., Byrne K., White J., McWilliam S., Reverter A., Cockett N.E. (2007). Identification of a gene network contributing to hypertrophy in callipyge skeletal muscle. Physiol. Genomics.

[19] Xu T.S., Gu L.H., Sun Y., Zhang X.H., Ye B.G., Liu X.L. (2015). Characterization of MUSTN1 gene and its relationship with skeletal muscle development at postnatal stages in Pekin ducks. Genet. Mol. Res..

[20] Gersch R.P., Kirmizitas A., Sobkow L., Sorrentino G., Thomsen G.H., Hadjiargyrou M. (2012). Mustn1 is essential for craniofacial chondrogenesis during Xenopus development. Gene Expr. Patterns.

[21] Gersch R.P., Hadjiargyrou M. (2009). Mustn1 is expressed during chondrogenesis and is necessary for chondrocyte proliferation and differentiation in vitro. Bone.

[22] Krause M.P., Moradi J., Coleman S.K., D'Souza D.M., Liu C., Kronenberg M.S. (2013). A novel GFP reporter mouse reveals Mustn1 expression in adult regenerating skeletal muscle, activated satellite cells and differentiating myoblasts. Acta Physiol. (Oxf).

[23] Hadjiargyrou M. (2018). Mustn1: a developmentally regulated pan-musculoskeletal cell marker and regulatory gene. Int. J. Mol. Sci..

[24] Liu C., Gersch R.P., Hawke T.J., Hadjiargyrou M. (2010). Silencing of Mustn1 inhibits myogenic fusion and differentiation. Am. J. Physiol. Cell Physiol..

[25] Kim C.J., Singh C., Lee C., DiMagno K., O'Donnell M., Kaczmarek M. (2023). Mustn1 ablation in skeletal muscle results in increased glucose tolerance concomitant with upregulated GLUT expression in male mice. Physiol. Rep..

[26] Fu Y., Hao X., Shang P., Nie J., Chamba Y., Zhang B. (2025). MUSTN1 interaction with SMPX regulates muscle development and regeneration. Cell Prolif..

[27] Livak K.J., Schmittgen T.D. (2001). Analysis of relative gene expression data using real-time quantitative PCR and the 2(-Delta Delta C(T)) Method. Methods.

[28] Cristancho A.G., Lazar M.A. (2011). Forming functional fat: a growing understanding of adipocyte differentiation. Nat. Rev. Mol. Cell Biol..

[29] Hu Z., Xu H., Lu Y., He Q., Yan C., Zhao X. (2021). MUSTN1 is an indispensable factor in the proliferation, differentiation and apoptosis of skeletal muscle satellite cells in chicken. Exp. Cell Res..

[30] Jou P.C., Ho B.Y., Hsu Y.W., Pan T.M. (2010). The effect of Monascus secondary polyketide metabolites, monascin and ankaflavin, on adipogenesis and lipolysis activity in 3T3-L1. J. Agric. Food Chem..

[31] Mota de Sá P., Richard A.J., Hang H., Stephens J.M. (2017). Transcriptional regulation of adipogenesis. Compr. Physiol..

[32] Li Y., Goto T., Yamakuni K., Takahashi H., Takahashi N., Jheng H.F. (2016). 4-Hydroxyderricin, as a PPARγ agonist, promotes adipogenesis, adiponectin secretion, and glucose uptake in 3T3-L1 cells. Lipids.

[33] Josephrajan A., Hertzel A.V., Bohm E.K., McBurney M.W., Imai S.I., Mashek D.G. (2019). Unconventional secretion of adipocyte fatty acid binding protein 4 is mediated by autophagic proteins in a Sirtuin-1-dependent manner. Diabetes.

[34] Sha R.S., Kane C.D., Xu Z., Banaszak L.J., Bernlohr D.A. (1993). Modulation of ligand binding affinity of the adipocyte lipid-binding protein by selective mutation. Analysis in vitro and in situ. J. Biol. Chem..

[35] Ghaben A.L., Scherer P.E. (2019). Adipogenesis and metabolic health. Nat. Rev. Mol. Cell Biol..

[36] Rosen E.D., MacDougald O.A. (2006). Adipocyte differentiation from the inside out. Nat. Rev. Mol. Cell Biol..

[37] Moseti D., Regassa A., Kim W.K. (2016). Molecular regulation of adipogenesis and potential anti-adipogenic bioactive molecules. Int. J. Mol. Sci..

[38] Gregoire F.M., Smas C.M., Sul H.S. (1998). Understanding adipocyte differentiation. Physiol. Rev..

[39] Yang X., Zhang X., Heckmann B.L., Lu X., Liu J. (2011). Relative contribution of adipose triglyceride lipase and hormone-sensitive lipase to tumor necrosis factor-α (TNF-α)-induced lipolysis in adipocytes. J. Biol. Chem..

[40] Yazıcı D., Sezer H. (2017). Insulin resistance, obesity and Lipotoxicity. Adv. Exp. Med. Biol..

[41] Tchkonia T., Thomou T., Zhu Y., Karagiannides I., Pothoulakis C., Jensen M.D. (2013). Mechanisms and metabolic implications of regional differences among fat depots. Cell Metab..

[42] Jiao Y., Liang X., Hou J., Aisa Y., Wu H., Zhang Z. (2019). Adenovirus type 36 regulates adipose stem cell differentiation and glucolipid metabolism through the PI3K/Akt/FoxO1/PPARγ signaling pathway. Lipids Health Dis..

[43] Bray G.A., Bellanger T. (2006). Epidemiology, trends, and morbidities of obesity and the metabolic syndrome. Endocrine.

[44] Hotamisligil G.S. (2006). Inflammation and metabolic disorders. Nature.

[45] Ullah M., Stich S., Häupl T., Eucker J., Sittinger M., Ringe J. (2013). Reverse differentiation as a gene filtering tool in genome expression profiling of adipogenesis for fat marker gene selection and their analysis. PLoS One.

[46] Ducommun S., Jannig P.R., Cervenka I., Murgia M., Mittenbühler M.J., Chernogubova E. (2024). Mustn1 is a smooth muscle cell-secreted microprotein that modulates skeletal muscle extracellular matrix composition. Mol. Metab..

[47] Kershaw E.E., Flier J.S. (2004). Adipose tissue as an endocrine organ. J. Clin. Endocrinol. Metab..

[48] Aguilar-Valles A., Inoue W., Rummel C., Luheshi G.N. (2015). Obesity, adipokines and neuroinflammation. Neuropharmacology.

[49] Antuna-Puente B., Feve B., Fellahi S., Bastard J.P. (2008). Adipokines: the missing link between insulin resistance and obesity. Diabetes Metab..

[50] Fantuzzi G. (2005). Adipose tissue, adipokines, and inflammation. J. Allergy Clin. Immunol..

[51] Leal Vde O., Mafra D. (2013). Adipokines in obesity. Clin. Chim. Acta.

[52] Balistreri C.R., Caruso C., Candore G. (2010). The role of adipose tissue and adipokines in obesity-related inflammatory diseases. Med. Inflamm..

[53] Fain J.N., Madan A.K., Hiler M.L., Cheema P., Bahouth S.W. (2004). Comparison of the release of adipokines by adipose tissue, adipose tissue matrix, and adipocytes from visceral and subcutaneous abdominal adipose tissues of obese humans. Endocrinology.

[54] Samulin J., Berget I., Lien S., Sundvold H. (2008). Differential gene expression of fatty acid binding proteins during porcine adipogenesis. Comp. Biochem. Physiol. B Biochem. Mol. Biol..

[55] Ma Z., Luo N., Liu L., Cui H., Li J., Xiang H. (2021). Identification of the molecular regulation of differences in lipid deposition in dedifferentiated preadipocytes from different chicken tissues. BMC Genomics.

[56] Del Collado M., da Silveira J.C., Sangalli J.R., Andrade G.M., Sousa L., Silva L.A. (2017). Fatty acid binding protein 3 and transzonal projections are involved in lipid accumulation during in vitro maturation of bovine oocytes. Sci. Rep..

[57] Yi B., Wang J., Wang S., Yuan D., Sun J., Li Z. (2014). Overexpression of Banna mini-pig inbred line fatty acid binding protein 3 promotes adipogenesis in 3T3-L1 preadipocytes. Cell Biol. Int..

[58] Jiang Y., Liu J., Liu H., Zhang W., Li X., Liu L. (2022). miR-381-3p inhibits intramuscular fat deposition through targeting FABP3 by ceRNA regulatory network. Biology (Basel).

[59] Li X., Kim S.W., Choi J.S., Lee Y.M., Lee C.K., Choi B.H. (2010). Investigation of porcine FABP3 and LEPR gene polymorphisms and mRNA expression for variation in intramuscular fat content. Mol. Biol. Rep..

[60] Chmurzynska A., Szydlowski M., Stachowiak M., Stankiewicz M., Switonski M. (2007). Association of a new SNP in promoter region of the porcine FABP3 gene with fatness traits in a polish synthetic line. Anim. Biotechnol..

[61] Cho K.H., Kim M.J., Jeon G.J., Chung H.Y. (2011). Association of genetic variants for FABP3 gene with back fat thickness and intramuscular fat content in pig. Mol. Biol. Rep..

[62] Wang H., Wang J., Yang D.D., Liu Z.L., Zeng Y.Q., Chen W. (2020). Expression of lipid metabolism genes provides new insights into intramuscular fat deposition in Laiwu pigs. J. Anim. Sci..

[63] Huang Z.G., Xiong L., Liu Z.S., Qiao Y., Liu S.R., Ren H.X. (2006). The developmental changes and effect on IMF content of H-FABP and PPARgamma mRNA expression in sheep muscle. Yi Chuan Xue Bao.

[64] Wang L., Li L., Jiang J., Wang Y., Zhong T., Chen Y. (2015). Molecular characterization and different expression patterns of the FABP gene family during goat skeletal muscle development. Mol. Biol. Rep..

[65] Zhang Y., Kent J.W., Lee A., Cerjak D., Ali O., Diasio R. (2013). Fatty acid binding protein 3 (fabp3) is associated with insulin, lipids and cardiovascular phenotypes of the metabolic syndrome through epigenetic modifications in a Northern European family population. BMC Med. Genomics.

[66] Kim Y.B., Uotani S., Pierroz D.D., Flier J.S., Kahn B.B. (2000). In vivo administration of leptin activates signal transduction directly in insulin-sensitive tissues: overlapping but distinct pathways from insulin. Endocrinology.

[67] Sharma B.R., Kim H.J., Rhyu D.Y. (2015). Caulerpa lentillifera extract ameliorates insulin resistance and regulates glucose metabolism in C57BL/KsJ-db/db mice via PI3K/AKT signaling pathway in myocytes. J. Transl. Med..

[68] Xia X., Serrero G. (1999). Inhibition of adipose differentiation by phosphatidylinositol 3-kinase inhibitors. J. Cell Physiol..

