# Supplementary material for: MUSTN1 and FABP3 interact to regulate adipogenesis and lipid deposition
Source: J Lipid Res. 2025 Apr 15;66(5):100804. doi: 10.1016/j.jlr.2025.100804 (PMC12139245; doi:10.1016/j.jlr.2025.100804)
Supplement: Supplemental material [file mmc1.docx]

**SUPPLEMENTAL MATERIALS**


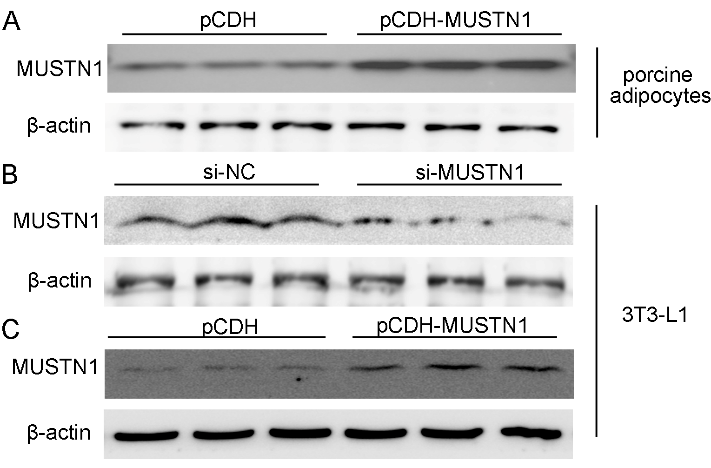


**Supplementary Figure S1 WB analysis of MUSTN1 overexpression and knockdown efficiency.** **A** MUSTN1 protein level in control or *MUSTN1* overexpressed porcine adipocytes. Protein detection for *MUSTN1* knockdown (**B**) or overexpression (**C**) efficiency in 3T3-L1.


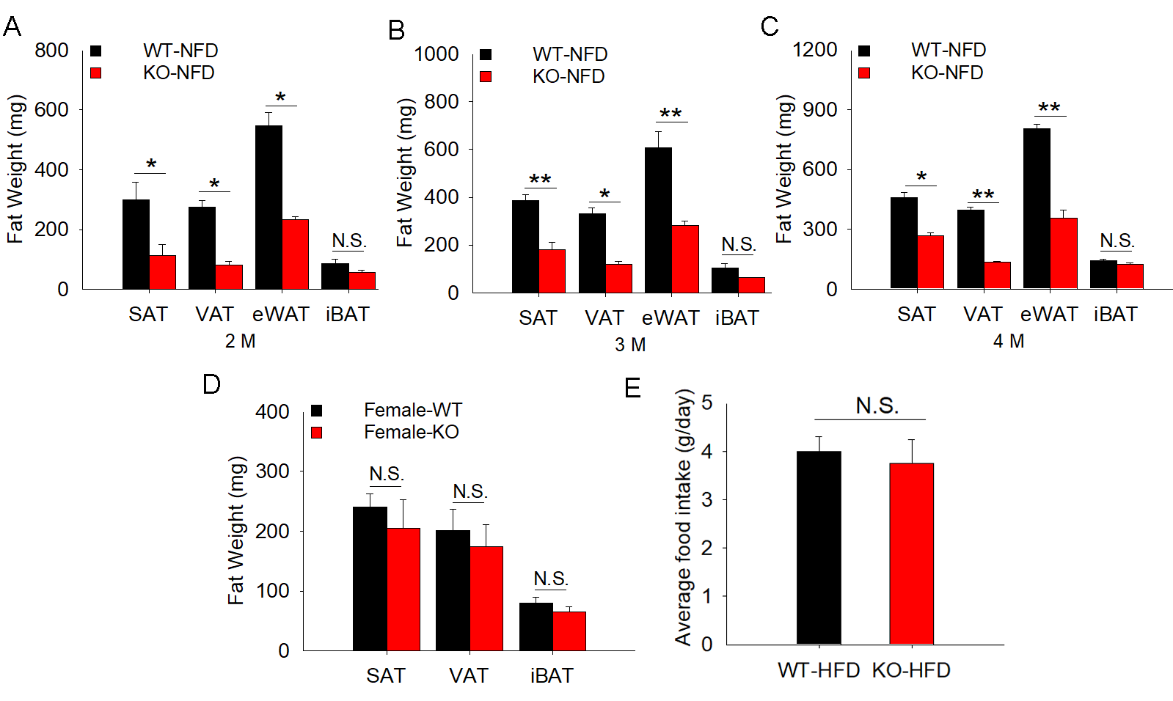


**Supplementary Figure S2 *MUSTN1*-KO mice showed decreased fat mass.** **A-C** Weight of WT and Tg mice aged from two to four months (2-4 M) on NFD. **D** Fat weight of normally fed 2-month-old female WT and KO mice. **E** Food intake between WT and KO mice. SAT: subcutaneous adipose tissue; VAT: visceral adipose tissue; eWAT: epididymal white adipose tissue; iBAT: interscapular brown adipose tissue. The data represent the mean ± SD of 6 independent experiments. *: *P*<0.05, **: *P*<0.01, as assessed by a student’s t-test. N.S.: not significant.


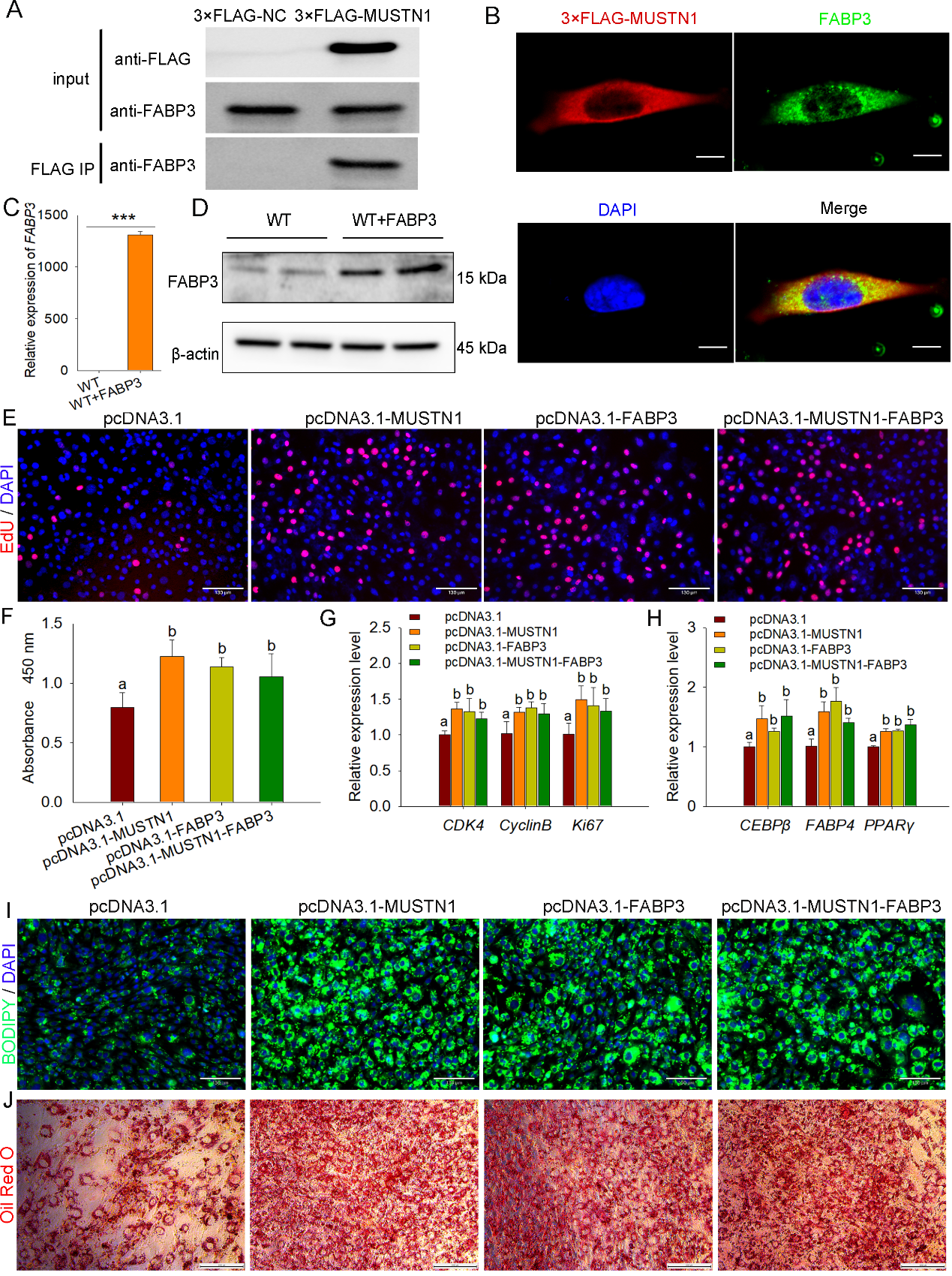


**Supplementary Figure S3 MUSTN1 and FABP3 had no synergistic effect in promoting adipocyte proliferation and differentiation.** **A** Reciprocal co-immunoprecipitation analysis between FLAG-tagged mouse MUSTN1 in 3T3-L1 cells. IP, immunoprecipitation. **B** Immunofluorescence staining for FABP3 and FLAG-tag in 3T3-L1 cells transfected with MUSTN1-3×FLAG plasmid, scale bar = 10 μm. The efficiency of FABP3 overexpression quantified by qRT-PCR (**C**) and WB (**D**). Representative photographs of EdU staining (**E**) and CCK8 statistical analysis (**F**). pcDNA3.1-*MUSTN1*-mouse and pcDNA3.1-*FABP3*-mouse were transfected and co-transfected into 3T3-L1 cells; scale bar, 130 μm. qRT-PCR analysis of proliferation (**G**) and differentiation (**H**) markers expression. Adipose differentiation analysis using BODIPY (**I**) and Oil Red staining (**J**). 3T3-L1 cells were treated with separate- and co-transfection of *MUSTN1* and *FABP3*, and differentiated for 8 days. BODIPY- and Oil Red O-stained fat droplets are shown in green and red, respectively; scale bar = 130 μm (BODIPY), 200 μm (Oil Red O). The data represent the mean ± SD of three independent experiments. Different letters represent significant differences based on Student’s *t*-test.

**Supplementary Table S1 The primer sequences for overexpression plasmid construction**

| Primer name | Primer sequences (5′ to 3′) |
| --- | --- |
| pCDH-*MUSTN1*-pig  pCDH-*MUSTN1*-mouse | F: ttcgaatttaaatcgCCACCATGTCCCAGGAA |
|  | R: tcgcagatccttcgcCACTTCTCAGCCAAAGACACT  F: ttcgaatttaaatcgATGTCCGAGGCTGGCACTCC  R: tcgcagatccttcgcGCCAAACACGCTCTTGGCTG |
| 3×FLAG-*MUSTN1-*mouse  3×FLAG-*FABP3*-mouse | F:tagcgtttaaacttaagcttgccaccATGTCCGAGGCTGGCACTCC |
|  | R: gctggatatctgcagaattcGCCAAACACGCTCTTGGCTG  F:tagcgtttaaacttaagcttgccaccATGGCGGACGCCTTTGTCGGTA  R: gctggatatctgcagaattcCGCCTCCTTCTCATAAGTCCGA |

**Supplementary Table 2. The sequences of *MUSTN1* siRNA fragments**

| siRNA (mouse) | Oligo sequences (5′ to 3′) |
| --- | --- |
| si-NC | CGAGCUCGAAUCACGGUCATT |
|  | UCAGGCUGAAGACCGGAUCTT |
| si-430 | GAGGGACCCUGGCCAAGAATT |
|  | UUCUUGGCCAGGGUCCCUCTT |
| si-579 | GCCAAGAGCGUGUUUGGCUTT |
|  | AGCCAAACACGCUCUUGGCTT |
| si-723 | GCCCUUGGAAACACCAAUATT |
|  | UAUUGGUGUUUCCAAGGGCTT |

**Supplementary Table S3.** **The primer sequences for SqRT-PCR and qPCR**

| Species | Genes | | Primer sequences (5′ to 3′) |
| --- | --- | --- | --- |
| Pig  Mouse | *MUSTN1*  *Ki67*  *CDK4*  *Cyclin B*  *BAD*  *PPARγ*  *CEBPβ*  *FABP4*  *FABP3*  *β-actin*  *PPARγ*  *CEBPα*  *CEBPβ*  *FABP4*  *Cyclin B* | F: CAGGAAGCCCCCATCAAGA  R: AGTCCGAGCACGGCTGAAC  F: ATCATTGACCGCTCCTTTAGGT  R: GCTCGCCTTGATGGTTCCT  F: CTTTGACCTGATTGGGCTGC  R: CAGAGATTCGCTTGTGTGGGT  F: TGAGGAAGAACAAGCAGTTAGACC  R: TCACAAAGGCAAAGTCACCAAT  F: TGAGCAGAGTGAGCAGGAAGAC  R: TGGGTAAGAGCTGTGGCGA  F: ACCAAAGCAAAGGCGAGG  R: GCGAAACTGACACCCCTGA  F: CCTGTCCACATCCTCGTCGT  R: CTCGTCGCTGTGCTTATCCA  F: CAGGAAAGTCAAGAGCACCA  R: GGTAGCCGTGACACCTTTC  F: AAAGCACCTTCAAGAGCACAGA  R: ACAAGTTTGCCTCCATCCAGT  F: GCCAACCGTGAGAAGATGACT  R: GTGACCCCATCCCCAGAGT  F: CCAAGAATACCAAAGTGCGATCA  R: CCCACAGACTCGGCACTCAAT  F: TGGACAAGAACAGCAACGAG  R: TCACTGGTCAACTCCAGCAC  F: ATCGACTTCAGCCCCTACCT  R: TAGTCGTCGGCGAAGAGG  F: AAGAAGTGGGAGTGGGCTTTG  R: CTCTTCACCTTCCTGTCGTCTG  F: ATACCTACAGGGTCGTGAAGTGA  R: GCTGTATCATCTTCTTGGGCAC | |
|  | *CDK4*  *Ki67*  *Fasn* | F: GCTGCTACTGGAAATGCTGACC  R: AGCCTTGGGGGGAAACAGA  F: AATCCAACTCAAGTAAACGGGG  R: TTGGCTTGCTTCCATCCTCA  F: CACTGCATTGACGGCCGGGT  R: GGACAAGCCCAGGCTGCGAG | |
|  | *Adipoq*  *LPL*  *HSL*  *ATGL*  *TNFα*  *MUSTN1*  *β-actin* | F: GCCGCTTATGTGTATCGCTCAG  R: TTGCCAGTGCTGCCGTCATA  F: ATGCAGAAGCCCCCAGTCGC  R: GCCCCACTGGTTTCTGGATCCCA  F: CACACCTACTACACAAATCC  R: GGCATAGTAGGCCATAGCA  F: CCAACGCCACTCACATCTAC  R: CCTCAATAATGTTGGCACCTG  F: AGGGTCTGGGCCATAGAACT  R: CCACCACGCTCTTCTGTCTAC  F: CCCCTGTGAAGGAAGAAGACC  R: CGGTTGCGGCTGAATACA  F: ATCTGGCACCACACCTTCTACA  R: AAGGTCTCAAACATGATCTGGGT | |

**Supplementary Table S4 The gRNAs and primers for *MUSTN1*^-/-^ mouse construction**

| Name | Sequences (5′ to 3′) |
| --- | --- |
| gRNA-1  gRNA-2 | ACAGCTGCTTACTCGACGGTGGG |
|  | ACACGTAATGCAATGCCCGCAGG |
| KO-F1  WT-F2  R1/R2 | GCTAAACAAACTCAGAGCATCCAC |
|  | ATAAAGAGTATGCCCAATGTCCCC |
|  | AAGGAGTAAGGATAATGGGCTTGG |
